# Assessing Photostability of mAb Formulations In Situ Using Light-Coupled NMR Spectroscopy

**DOI:** 10.1021/acs.analchem.4c01164

**Published:** 2024-06-07

**Authors:** Jack E. Bramham, Yujing Wang, Stephanie A. Moore, Alexander P. Golovanov

**Affiliations:** † Department of Chemistry, School of Natural Sciences, Faculty of Science and Engineering, The University of Manchester, Manchester M1 7DN, U.K.; ‡ Dosage Form Design & Development, BioPharmaceutical Development, R&D, AstraZeneca, Cambridge CB2 0AA, U.K.

## Abstract

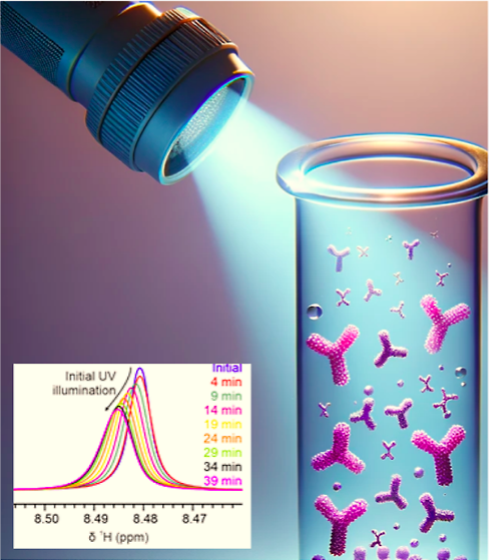

Biopharmaceuticals, such as monoclonal antibodies (mAbs), need to maintain their chemical and physical stability in formulations throughout their lifecycle. It is known that exposure of mAbs to light, particularly UV, triggers chemical and physical degradation, which can be exacerbated by trace amounts of photosensitizers in the formulation. Although routine assessments of degradation following defined UV dosages are performed, there is a fundamental lack of understanding regarding the intermediates, transient reactive species, and radicals formed during illumination, as well as their lifetimes and immediate impact post-illumination. In this study, we used light-coupled NMR spectroscopy to monitor in situ live spectral changes in sealed samples during and after UV-A illumination of different formulations of four mAbs without added photosensitizers. We observed a complex evolution of spectra, reflecting the appearance within minutes of transient radicals during illumination and persisting for minutes to tens of minutes after the light was switched off. Both mAb and excipient signals were strongly affected by illumination, with some exhibiting fast irreversible photodegradation and others exhibiting partial recovery in the dark. These effects varied depending on the mAb and the presence of excipients, such as polysorbate 80 (PS80) and methionine. Complementary ex situ high-performance size-exclusion chromatography analysis of the same formulations post-UV exposure in the chamber revealed significant loss of purity, confirming formulation-dependent degradation. Both approaches suggested the presence of degradation processes initiated by light but continuing in the dark. Further studies on photoreaction intermediates and transient reactive species may help mitigate the impact of light on biopharmaceutical degradation.

## Introduction

Due to the growing importance of biopharmaceuticals, such as monoclonal antibodies (mAbs), in treating various diseases, it is crucial to understand triggers and mechanisms of their degradation in order to develop safe and efficacious final dosage forms.^1,2^ Among the triggers of biopharmaceutical instabilities, the effects of light are understudied.^3^ Both the active pharmaceutical ingredient (API) itself and small-molecule formulation excipients may be degraded by ultraviolet (UV) and/or visible light.^4−6^ Although most biopharmaceutical production and handling takes place indoors, modern lighting can still contain significant amounts of blue and, to a lesser extent, near UV (300–400 nm) light, for example, from fluorescent lamps and LEDs, resulting in photodegradation.^7,8^

The mechanisms and pathways of biopharmaceutical photodegradation are numerous and complex. While aromatic residues absorb light in the UV–B and −C wavelengths (<320 nm), resulting in direct protein photodegradation,^9,10^ biopharmaceutical formulations are not exposed to such wavelengths under typical indoor lighting (320–700 nm). However, various molecules acting as photosensitizers present in biopharmaceutical formulations, for example, histidine (His) buffer degradation products,^5,11,12^ riboflavin contaminants from cell culture,^13^ citrate buffer-Fe^3+^ complexes,^14,15^ or polysorbates,^16,17^ may absorb UV-A or visible light and trigger protein degradation via direct reaction of photosensitizers with protein residues or via formation and subsequent action of reactive oxygen species (ROS).^18^ Additionally, oxidation products of tryptophan^19,20^ or tyrosine^21^ residues absorb UV-A light, potentially promoting further protein degradation. The result of these processes is chemical modification of the protein,^22^ which can reduce bioactivity^23,24^ and trigger physical degradation such as aggregation and fragmentation,^25,26^ product discolouration,^20,27,28^ as well as immunogenic issues.^29^ Photodegradation of excipients, such as histidine^5,11,12^ and polysorbates,^17,30^ alongside generating photosensitizers, may destabilize the formulation against other stresses which the excipients usually protect against. Alternatively, additives, for example, antioxidants like methionine (Met), may be included in formulations acting as “sacrificial” excipients, specifically undergoing oxidation themselves to minimize API degradation.^31,32^ Characterizing all of these components and their response to illumination is therefore important to determine the appropriate controls required regarding light exposure during manufacturing and fill-finish operations as well as during administration. To create accelerated stressed conditions for photostability testing, photosensitizers may be added, e.g., hydrogen peroxide to force oxidation;^33,34^ however, such additions may mask the effects of the traces of endogenous photosensitizers inherent to formulations.

Due to the recognized importance of photostability for all pharmaceuticals, guidelines exist drawn by the International Council for Harmonization of Technical Requirements for Pharmaceuticals for Human Use (ICH) Q1B, which define light exposure limits under which the drug product should remain stable–visible light ≥1.2 million lux hours and integrated UV energy ≥200 W h m^–2^.^35,36^ However, analytical measurements are typically made only before and after light exposure in an illumination chamber, potentially missing transient processes and short-lived intermediate components, such as ROS, present in solution both during and immediately after illumination. To better understand photodegradation and subsequent degradation, it would be beneficial to monitor and characterize the process in situ for the entire formulation without any sample modifications or dilution, which may distort analysis.

We previously demonstrated that NMR spectroscopy can detect and provide a holistic overview of both API and excipient degradation in situ in high-concentration formulations.^37^ Here, we demonstrate that this NMR-based holistic approach can be extended to monitor the accelerated degradation of mAb formulations when illuminated in situ using high-intensity UV-A light with the NMRtorch approach.^38,39^ Exposure to strong UV light, even for a relatively short time, triggers a chain of degradation events that continue even in the dark, which can all be monitored by light-coupled NMR spectroscopy. The complementary experiments performed with UV light chamber illumination, with regular taking out aliquots for high-performance size-exclusion chromatography (HP-SEC) analysis, confirm significant degradation of mAbs, which is formulation and mAb dependent.

## Experimental Section

### Sample Preparation and Photodegradation Studies in UV Chamber

Four antibodies (mAbs) were supplied by AstraZeneca and included IgG1 (mAb1, mAb2, and mAb4) and bispecific (Bs-Ab3) types. The mAbs were extensively dialyzed over 3 days into 20 mM histidine (His) buffer pH 5.5 (l-histidine and l-histidine monohydrochloride monohydrate, both Avantor, #2080-06 and #2081-06, respectively) using GeBAflex Midi dialysis tubes (Generon, 8 kDa MWCO), concentrated using Vivaspin 20 (30 kDa MWCO, Sartorius), and formulated to 40 mg/mL. Various mAb formulations were prepared, with or without 0.1% polysorbate 80 (PS80, Avantor #4117) or 20 mM Met (Merk #M9625). The samples were 0.22 μm filtered before placing 0.5 mL of each into 2R type 1 borosilicate glass vials. The vials, together with “dark control” samples of each mAb in 20 mM His buffer wrapped in aluminum foil, were placed in a UV chamber (Caron, model 7545-11-3, UV-A exposure of 30 W/m^2^, temperature of 20 °C and humidity of 40%). The vials were exposed for up to 7.5 h, after which the samples remained in the chamber for a further 16.5 h to explore further degradation under darkness. An aliquot of 25 μL of each sample was removed from the vials for high-performance size-exclusion chromatography (HP-SEC) analysis at 0, 1, 2.5, 4, 5.5, 7.5, and 24 h time points. An independent calibration experiment was conducted and determined the equivalent OD change of a 2% quinine solution at 400 nm throughout the same time points (Supporting Information Figure S1).

### High-Performance Size-Exclusion Chromatography

Analysis of mAb monomeric, high molecular weight (HMW) species, and lower molecular weight (LMW) species was performed using an Agilent 1260 series HPLC system with a TSKgel SWXL column (30 cm × 7.8 mm, 5 μm particle size, Tosoh Bioscience). The formulations were diluted to 10 mg/mL and 0.45 μm filtered prior to analysis (Ultrafree-MC-HV, Merck Millipore). A 25 μL injection volume was used, run at 1.0 mL/min with a mobile phase of 0.1 M Na_2_HPO_4_, 0.1 M Na_2_SO_4_, pH 6.8. Chromatograms (detection at 280 nm) were analyzed in ChemStation (Agilent), with concentration of species calculated as percentages. The results of the monomer, LMW, and HMW species analysis of the dark control samples throughout the time course were averaged for each mAb, and the standard deviation (SD) was calculated as an estimate for the baseline degradation level and method variability. In illuminated samples, changes greater than 3 × SD were then considered a “meaningful” change (0.05, 0.27, 0.05, and 0.01% for mAb1, mAb2, mAb3, and mAb4, respectively).

### Sample Preparation for Light-Coupled NMR Studies

For NMR experiments, the original mAb stocks were extensively dialyzed over 3 days against 20 mM His buffer, pH 5.5 (l-histidine and l-histidine monohydrochloride monohydrate, both Sigma-Aldrich, #H6034 and #H5659, respectively) using GeBAflex Midi dialysis tubes (Generon, 8 kDa MWCO). Samples were filtered with 0.22 μm filters (PVDF, Merck Millipore) and concentrated using a Vivaspin 500 (30 kDa MWCO, Sartorius). Protein concentration was determined by using absorbance at 280 nm using known extinction coefficients and a NanoDrop spectrometer (ThermoScientific).

Final 40 mg/mL mAb samples for NMR were supplemented with 5% ^2^H_2_O for lock and with the further addition of either 0.1% PS80 (P1754, Sigma-Aldrich) or 20 mM Met (M5308, Sigma-Aldrich). Samples of His buffer without mAbs and His buffer with 0.1% PS80 were also prepared and analyzed.

### In Situ Sample Illumination Using NMRtorch

UV-A illumination was performed using an NMRtorch lighthead with an array of 4 × 365 nm LEDs (LZ4-V4UV0R, LED Engin, nominal power 10 W), same as in previous studies,^38,39^ with illumination controlled by the NMR console and transistor–transistor logic triggers. Samples were placed in 5 mm quartz NMRtorch tubes, prepared as previously described,^38,39^ and sealed with transparent caps made from hand-polished 7 mm long Suprasil rods (Heraeus) and pieces of fluorinated ethylene propylene (FEP) tubing with 4.5 mm inner diameter.

### NMR Spectroscopy

^1^H NMR experiments were conducted using a Bruker 800 MHz AVANCE III spectrometer with a 5 mm TCI cryoprobe and variable temperature control unit. Experiments were acquired at 40 °C, with samples equilibrated for 10 min before shimming. A set of general characterization experiments were acquired before illumination: 1D ^1^H NMR spectra (*p3919gp* and *zgesgp*), transverse relaxation (T_2_)-filtered ^1^H spectra (150 ms T_2_ filter to remove fast relaxing mAb signals), diffusion ordered spectroscopy (DOSY) experiments (*stebpgp1s19pr*, with 300 ms diffusion time and 3 ms gradient pulses), a Carr–Purcell–Meiboom–Gill experiment (800 μs fixed echo time, with up to 128 CPMG loops) for determination of T_2_ times, and an inversion recovery experiment (with delays varying from 0.001 to 3 s in 10 steps) for determination of longitudinal relaxation times (T_1_). After recording this initial set of spectra for each sample in the darkness, the UV-A light was switched on to illuminate the samples in situ for 2 h, followed by light off (darkness) for a further 4 h, with 1D (*p3919gp*, 64 scans, 1.6 s relaxation delay) and T_2_-filtered spectra (40 scans, 1.5 relaxation delay) recorded in an interleaved manner at 5 min intervals throughout illumination and darkness, with 1.02 s acquisition time. Uncertainty in chemical shift measurement of His signals was around 0.25 Hz. Finally, the suite of general characterization experiments was recorded again. A marginal sample heating occurred during illumination which led to ∼2.7 Hz or ∼0.003 ppm spectral shift, equivalent to <0.5 °C raise which required ∼2 min sample re-equilibration after light switching; this spectral shift was constant throughout illumination, therefore the spectra acquired during illumination were rereferenced using position of dominant mAb methyl signals. Internal reference standards were not used to avoid their interactions with the formulation components. NMR data processing was performed in TopSpin 4.1.4 (Bruker), with diffusion and relaxation experiments analyzed using Dynamics Center 2.7.1 (Bruker). Graphs were drawn in Excel 2016 (Microsoft) and Prism 9.2.0 (GraphPad), with final figures prepared in CorelDRAW 2020 (Corel).

## Results and Discussion

### Studies of mAb Photodegradation by HP-SEC

**Figure 1:**
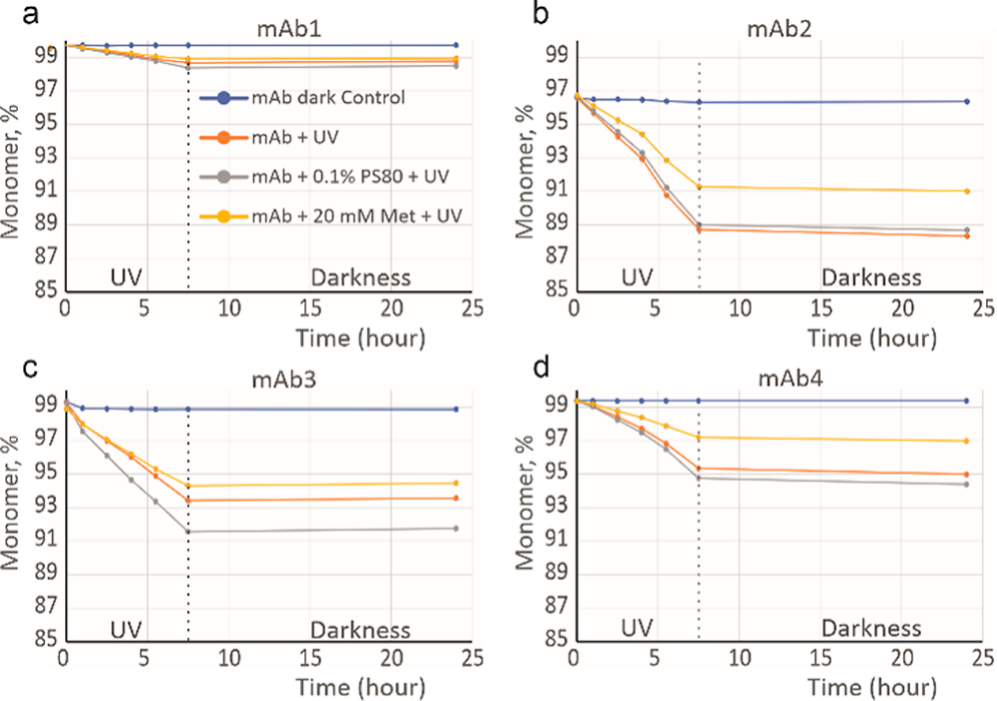
UV stress studies of mAb solutions analyzed by HP-SEC. Percentage of soluble monomer is shown for mAb1 (a), mAb2 (b), mAb3 (c), and mAb4 (d). Dark control samples were kept in a UV chamber but wrapped in aluminum foil to prevent UV exposure. All solutions contained 20 mM His buffer (pH 5.5), without or with excipients added as shown. After 7.5 h, UV light was switched off, with another aliquot taken for analysis at 24 h to explore further changes in the darkness.

First, we explored how UV-A illumination stress affects the percentage of mAb monomer present in solution, as well as amounts of HMW, i.e., aggregate, and LMW, i.e., fragment, species, using HP-SEC. Formulations of four mAbs in His buffer, with or without addition of PS80 or Met excipients, were placed in a UV chamber, and aliquots were analyzed by HP-SEC at regular intervals during illumination and also after the following dark period. Light-dependent degradation and the degradation continuing in the darkness, compared with control samples of the same mAbs protected from light, were monitored. All mAb formulations showed time-dependent degradation upon UV-A exposure. MAb1 appears to be the most stable, showing the least overall decrease in purity upon UV exposure (Figures 1a, S2a and S3a). Relative to the other three mAbs, the presence of Met or PS80 in the formulation has the least impact on the overall aggregation or fragmentation propensity of mAb1. The presence of PS80 in the formulation of mAb1 causes a greater loss of monomer compared to just the His and His and Met formulations. The formulation containing Met results in the least loss of monomer. No significant change in degradation state of mAb1 post-illumination between 7.5 and 24 h (when the UV chamber lights are switched off) is observed. For mAb2, the drop in monomer content upon illumination is much more prominent and is accompanied by notable increases in the rate of both aggregation and fragmentation (Figures 1b, S2b and S3b). For mAb2, the rate of UV-induced aggregation is reduced by the presence of Met; Met, however, has no impact on fragmentation. There are no meaningful stabilizing or destabilizing effects of PS80. Interestingly, the degradation profiles of mAb2 show a marginal trend for continued degradation even after the UV light is switched off, compared with the dark control sample (Figures 1b, S2b and S3b). The degradation of mAb3 is primarily driven by aggregation with no meaningful impact of UV on fragmentation (Figures 1c, S2c and S3c). For mAb3, the presence of Met decreases light-induced monomer loss and aggregation, whereas an increase in aggregation is observed in the PS80 formulation (Figures 1c and S2c). For mAb3, there is no degradation during the darkness period following the illumination; on the contrary, there is a noticeable recovery in monomer percentage (∼0.2%), which is mirrored by a loss of aggregate percentage (Figures 1c and S2c). This indicates that some of the aggregates formed during illumination dissociated in the darkness. The data for mAb4 suggests that degradation rate in response to UV illumination accelerates with time. Here, the primary degradation pathway for mAb4 is also aggregation (Figures 1d, S2d and S3d). The presence of Met in the formulation reduces the rate of light-induced aggregation, while PS80 appears to enhance it (Figure 1d). The degradation of mAb4 in all formulations appears to continue even when UV is switched off after 7.5 h as aggregate level increases by ∼0.3%, which is mirrored by a loss in monomer. Although small, these changes in the darkness following illumination for mAb2, mAb3, and mAb4 are nevertheless significant, compared to no changes in the respective dark control samples, suggesting continued evolution of the formulations after UV exposure.

Overall, the HP-SEC analysis revealed that even in the absence of any deliberately added photosensitizers, all four mAbs were susceptible to UV-induced degradation. The degradation rates varied between mAbs and formulations, but the predominant degradation pathway for all four molecules was aggregation. Common formulation excipients, PS80 and Met, had different effects on UV light-induced aggregation; overall, Met appears to reduce the rate of aggregation for all four mAbs, while the presence of PS80 mostly enhanced aggregation, except for mAb2 where the difference was small. These experiments also hinted at the presence of dark processes following the initial UV exposure (as with mAb3), where there may be dissociation of soluble aggregates back to monomeric species after relaxing in the dark. It is acknowledged that these preliminary observations would require further investigation. The observations imply the presence of light-induced species in the formulation that can modulate the degradation of mAbs. It should be noted that HP-SEC may not provide a quantitative assessment of fragmentation as not all fragments can be detected here, and other methods, such as gel electrophoresis, should be used for LMW quantitation. Additionally, HP-SEC may not fully capture changes in reversible self-association directly as the samples are diluted and buffer-exchanged during SEC runs. At the next stage, we continued with in situ NMR analysis of illuminated intact samples, so any observed changes in the signals from formulations are directly attributable to the effects of illumination.

### Monitoring Photodegradation of Formulations in Situ by Light-Coupled NMR Spectroscopy

**Figure 2:**
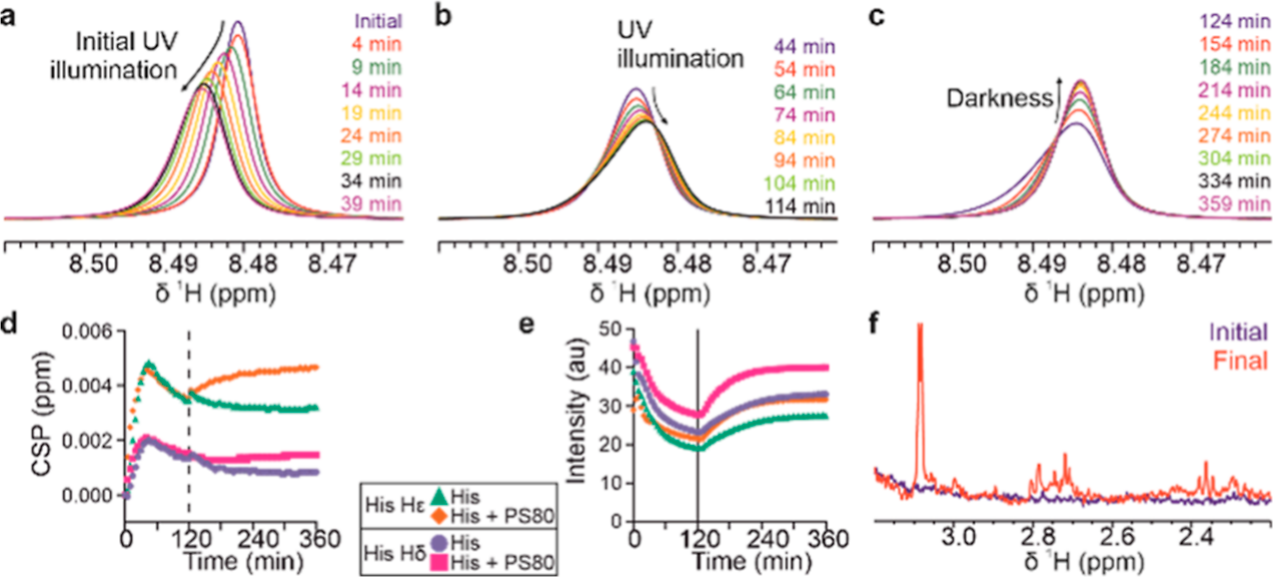
UV-A photodegradation of 20 mM His buffer. Appearance of the representative His Hε NMR signal during initial (a) and prolonged UV illumination (b) and during subsequent darkness (c). Evolution of the His Hδ and Hε chemical shift perturbation (d) and signal intensity (e). (f) New minor signals from degradation species created by illumination of a His buffer. Measurement uncertainty is smaller than the size of the symbols.

First, UV-A illumination was performed on His buffer alone and His with 0.1% PS80, both without mAbs, to characterize the effect of illumination on these components that potentially can act as endogenous photosensitizers. NMR spectra were recorded at 5 min intervals during 2 h of UV-A illumination, followed by a period of darkness for a further 4 h, to identify transient processes not usually captured by ex situ analytical methods. Here, both samples exhibited spectrum-wide changes upon illumination (Figure 2), with signals initially exhibiting chemical shift perturbations and broadening within the first 40 min (Figure 2a), before exhibiting even greater line shape distortions during the remaining UV illumination (up to 120 min, Figure 2b). After illumination was halted, the signals, noticeably their line shapes and chemical shifts, recovered slowly but did not return to their initial states, even after 4 h in the darkness (Figure 2d,e). These changes are not consistent with any effects of heating from illumination and instead likely indicate the formation of ROS or radicals by the UV-A light, with these transient reactive species perturbing the NMR properties and thus spectra of all molecules. The perturbation of observable NMR signals by these species is important to consider when assessing the effect of illumination in samples with mAbs. Additionally, illumination resulted in the formation of new detectable minor species with low signal intensity (Figure 2f), suggesting irreversible degradation of histidine by the emerging radical species. Together, these data show that, even in the absence of sensitizers added, the intense UV-A illumination results in the formation and accumulation of transient reactive species, which are then depleted fairly slowly during subsequent darkness. The presence of these transient species distorts the line shapes of NMR signals, changing shifts and line width, and likely causes chemical degradation of formulation components, which continues even after the light is switched off. The presence of PS80 in the formulation led to a different trajectory of movement of His signals in time after the illumination, suggesting that the nature and persistence of these transient radicals are formulation-dependent (Figure 2d,e). It should be noted that due to their paramagnetic properties and low concentrations, ROS and radicals themselves are not expected to be visible in NMR spectra, and therefore the presence of these reactive transient species here can be only inferred from their effects on observable NMR signals.

### Effect of UV-A Illumination on mAbs Studied by *NMR* in Situ

**Figure 3:**
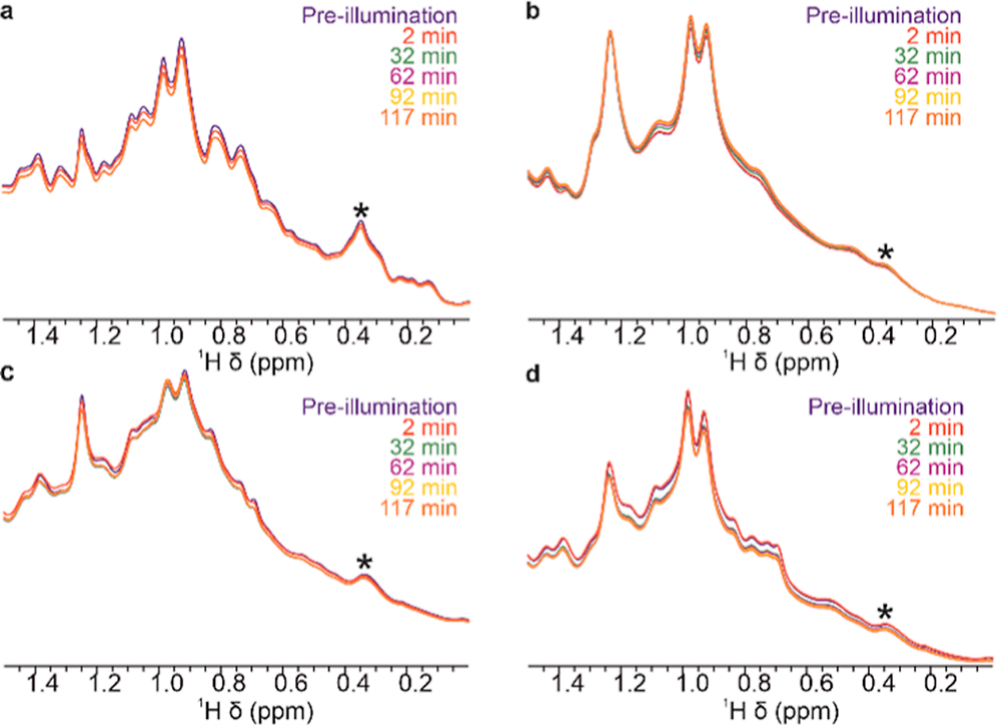
Changes in mAb NMR signals during UV illumination. (a) mAb1, (b) mAb2, (c) mAb3, and (d) mAb4. Representative mAb signals (at ∼0.35 ppm, selected due to no overlap with excipient signals) used for further analysis of degradation behavior are denoted with asterisks.

**Figure 4:**
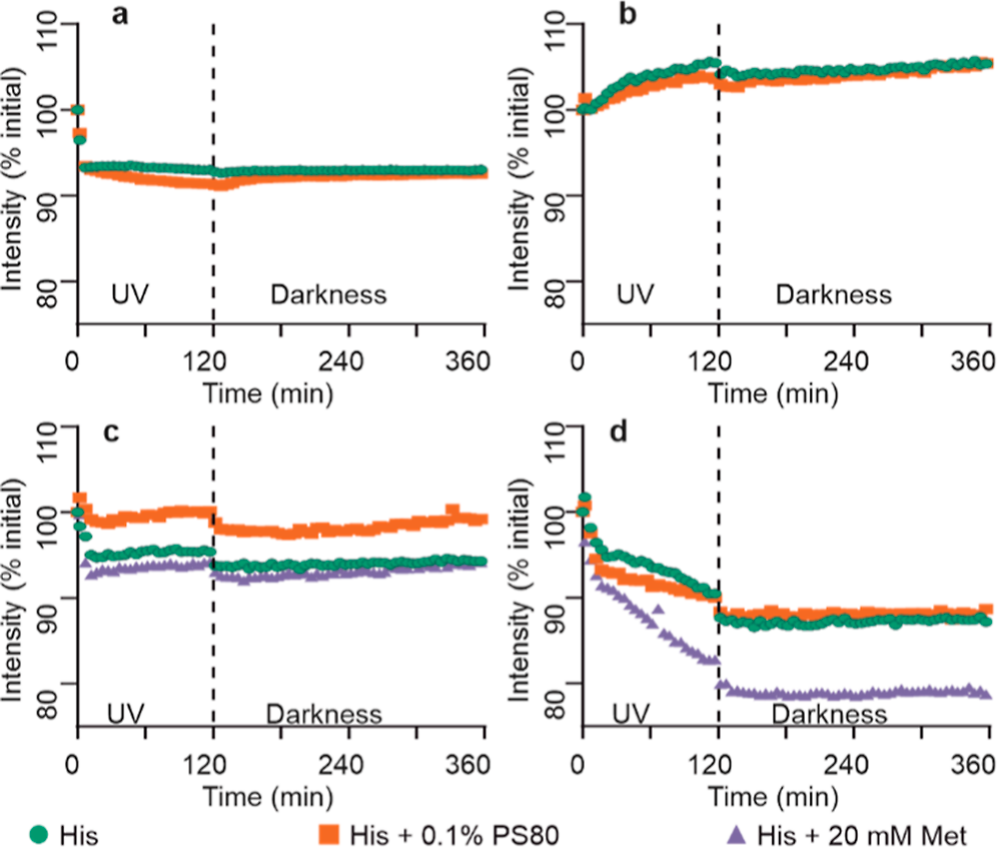
Changes in mAb representative NMR peak intensity during UV illumination and subsequent darkness. (a) mAb1, (b) mAb2, (c) mAb3, and (d) mAb4. Representative peaks were chosen in methyl spectral region at ∼0.35 ppm, typical S/N of >280.

Next, we characterized the effect of UV-A illumination on mAb formulations, focusing on the evolution of both protein and formulation component signals. Again, ^1^H NMR mAb signals demonstrate change during both UV illumination and the subsequent waiting period in the dark, indicating direct effects during the UV illumination, and prolonged effects which take time to manifest after the illumination is switched off. As mAb NMR spectral fingerprints differ between different mAbs, and the tendencies in changing the overall spectral intensities were consistent for each mAb, we chose one representative signal (marked with an asterisk on Figure 3) for each mAb to monitor its characteristic behavior, which were then plotted against time and illumination regime for selected formulations (Figure 4). Different mAbs clearly exhibit different behaviors in response to illumination. After the initial fast drop in signal intensity within first 10 min of illumination, mAb1 appears most stable with very little change in its characteristic signal intensity over time in the His buffer alone, with, however, more pronounced signal changes when PS80 was present (Figure 4a). MAb2 displays an overall gradual increase in signal intensity, without significant initial drop in signal intensity. MAb3 and mAb4 also show a quick initial drop in signal intensity, followed by a more gradual drop and more complex signal evolution (Figure 4).

As was discussed previously,^37^ if aggregation or self-association (i.e., formation of larger species with faster transverse relaxation rates R_2_) occurs, then the overall observed protein signal will reduce in intensity. Conversely, if fragmentation occurs (i.e., formation of smaller species with slower R_2_), then these faster-tumbling species would give rise to signals with larger intensity, while the shape of the overall mAb NMR signal may not change significantly due to lack of significant perturbation of the local 3D structure of fragments (e.g., F_ab_, F_c_, or intact Ig domains). As these two opposing processes may both occur simultaneously in a given sample, somewhat counteracting the others’ effect on the apparent signal intensities, mechanistic interpretation of this approach should be used with caution. Moreover, here, with illumination, signal intensity analysis may be further distorted by the transient presence of ROS and radicals, which may broaden and distort signals from mAbs and other formulation components, thus also decreasing signal intensity, making direct comparisons with HP-SEC profiles difficult. Nevertheless, here, mAb2 shows predominant tendency for signal increase during illumination, suggesting that protein fragmentation may be a significant pathway for its photodegradation. For mAb4 (Figure 4d), fast signal decrease is characteristic of significant protein aggregation and monomer loss. Two of the formulations, for mAb3 and mAb4, also had Met added as potentially sacrificial excipient to check if it would stabilize formulations against photodamage. In the presence of Met, the drop in characteristic signal intensities for mAb3 and mAb4 was actually more significant, more so for mAb4 (Figure 4c,d), suggesting that in the presence of Met and UV, the mAb signals get broadened, either due to increased protein self-association or due to higher presence of paramagnetic radicals.

Interestingly, in addition to differences for different mAbs in their general response to illumination followed by darkness, there are several common features in these profiles which are also indicative of the appearance of transient species which form in solution when the light is switched on and then persist for a while after the light is switched off. We link these effects with the possible appearance of ROS and radicals, which participate in further reactions before being eventually consumed after formulations are relaxed in the darkness. Appearance and evolution of such species can be more conveniently monitored by their effects on small-molecule components of mAb formulations—signals from His, PS80, and Met.

### Effect of UV-A Illumination on Small-Molecule Formulation Components of mAb Formulations

**Figure 5:**
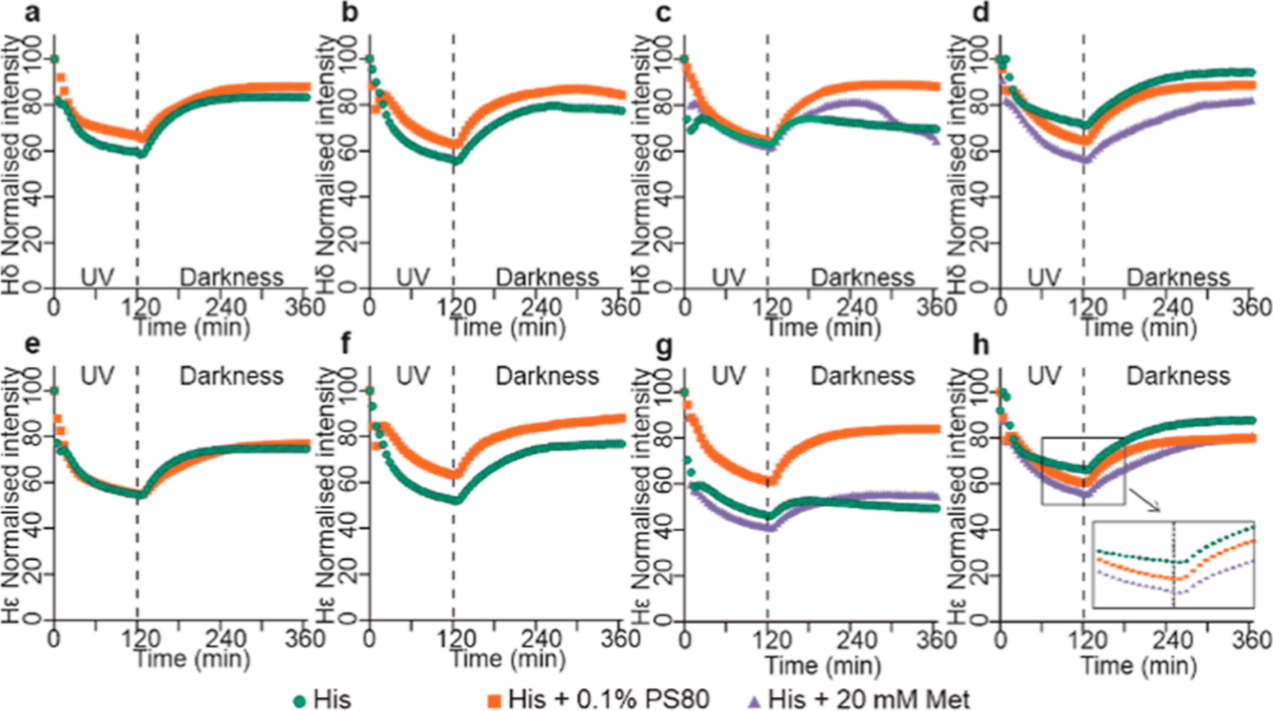
Changes in histidine buffer Hδ and Hε signal intensity during and after UV illumination. Hδ in (a) mAb1, (b) mAb2, (c) mAb3, and (d) mAb4 formulations. Hε in (e) mAb1, (f) mAb2, (g) mAb3, and (h) mAb4 formulations. Inset shows enlarged section of the graph around UV switch-off event. Signal intensity normalized against the initial value for each formulation. Typical S/N for His signals was >5000.

Histidine signals in all mAb formulations exhibit significant decrease in intensity (Figure 5) and line shape disturbances during illumination (Figure S4). During subsequent incubation in the darkness, signal line shapes mostly return to the expected Lorentzian shapes, but the final signals show marked change in chemical shift and reduced signal intensity when compared to the initial spectra (Figure S4). Overall, this process indicates the appearance of some form of transient paramagnetic species, i.e., ROS or radicals, created during illumination which perturb the NMR spectra (e.g., causing line shape distortion). During darkness, these species react away, resulting in less and less distortion of the NMR spectra with time—although notably, this process is slow over many minutes. The drops in signal intensity and the shape of its time dependence in different formulations and with different mAbs (Figure 5) suggest that these transient radical species are formulation-dependent. Interestingly, in the presence of Met, the drops in His signal intensities are most prominent (Figure 5c,d,g,h), as was also observed for mAb signals in these formulations (Figure 4c,d). This may indicate a larger concentration of radicals forming in the presence of Met, which may suggest that this sacrificial oxidation protectant is itself participating in transient radical formation under UV-A light. The characteristic time scales of appearance and disappearance of the reactive species are in the order of tens of minutes (Figure 5), implying that ex situ analysis of formulation content during and after the illumination may overlook the presence of these transient species. Interestingly, when the UV light is switched off at 120 min, the graphs (Figure 5) show a lag in signal response; instead of immediate start of signal recovery in the darkness, signal decay for some His signals still continues for a few minutes (e.g., Figure 5h). Some discontinuity following UV switch-off is observed for mAb signals as well (Figure 4) and cannot be explained by a small change in sample temperature. This, however, may be an indication of the complexity of transient reactive species interconversions and possibly the effect of these transient species on reversible mAb self-association, e.g., by modulating electrostatic interactions.

The presence of transient reactive radicals is expected to affect the T_1_ and T_2_ relaxation rates of the molecules present in solution. Measuring the evolution of such rates live throughout the in situ illumination experiment was impractical as such measurements are typically too long for the time scales involved. For this reason, these relaxation times were measured only before and after the illumination series, followed by darkness (at 0 and 360 min time points). The values of T_1_ for His signals systematically increased in the vast majority of cases (Figure S5), consistent with paramagnetic oxygen initially present in the samples being consumed. Such depletion of oxygen following illumination has been described previously.^4−6^ For T_2_ values of His signals, a borderline tendency for a decrease was observed for the end point, consistent with signal broadening (Figure S6). However, it should be noted that T_2_ relaxation times strongly depend on a number of factors other than paramagnetism, including aggregation in formulation, chemical exchange, and transient interactions. In the future, it would be interesting to measure the values of T_1_ and T_2_ relaxation times during the illumination, when the effect of paramagnetic transient reactive species would be at its maximum, and perform other types of NMR characterisations,^40,41^ provided that such experimental acquisitions will be fast enough to match the time scales of the reaction.

We also measured translational diffusion coefficients *D*_L_ for His signals before and after illumination to infer any changes in overall formulation viscosity following UV illumination (Figure S7). For the majority of mAb solutions, the changes in *D*_L_ were not significant, except for mAb3 formulations with His or His + PS80, where *D*_L_ was decreased significantly post-illumination, suggesting a light-induced increase in formulation viscosity for this bispecific antibody. Again, in the future, it would be useful to measure the diffusion during the illumination rather than in a relaxed solution after the stress, as it may help to disentangle the effect of increased viscosity on signal intensity from the effect of transient paramagnetic species on signal broadening and relaxation.

**Figure 6:**
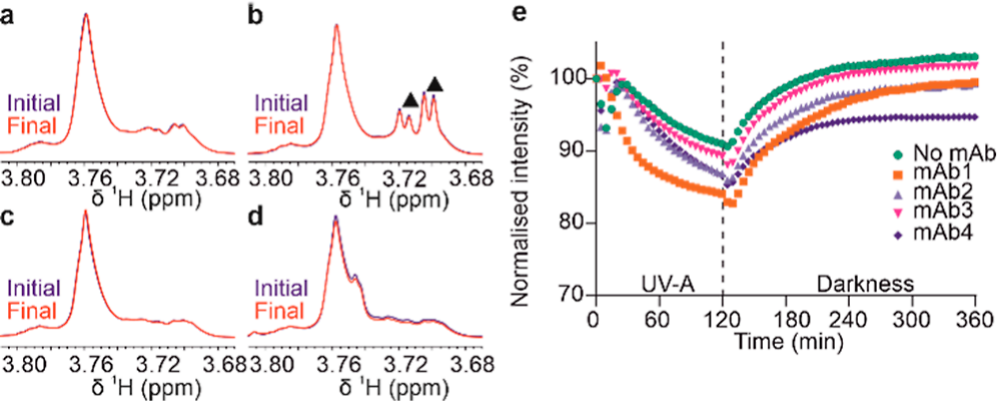
Appearance of PS80 signals in formulations with 0.1% PS80 before and after illumination. (a) mAb1, (b) mAb2 (with ▲ indicating overlapping signals from trace amounts of arginine remaining after extensive dialysis of the original stock mAb solutions), (c) mAb3, and (d) mAb4. (e) Changes in the PS80 NMR peak signal intensity (∼3.76 ppm) during illumination and darkness. Typical S/N for PS80 signals was >5000.

Polysorbates (such as PS80) are typically included in formulations to minimize interfacial stresses and degradation but are known to be particularly light sensitive.^42^ Previously, others have observed that while NMR can quantitatively determine polysorbate concentration in formulations, polysorbate degradation may only produce subtle, seemingly insignificant changes in polysorbate NMR signals.^43,44^ Here, PS80 signals in formulations of mAb1, mAb2, and mAb3 were indeed largely identical before and after UV-A illumination with following relaxation in the dark, but PS80 in the mAb4 formulation did show a ∼7% decrease in signal intensity at the final time point (Figure 6a–d). However, observing PS80 signal intensity in situ throughout the illumination and the following darkness revealed that signal intensities were changing very significantly and showed complex trajectories, depending on the type of mAb present (Figure 6e), with signals in different formulations recovered at the end except for mAb4. In the sample without any mAb, the PS80 signal showed initial dip in intensity during the illumination, likely caused by the production and consumption of ROS originating from oxygen initially dissolved in the sample. The depletion of oxygen initially present in a sample following illumination has been observed before.^4−6^ The time trajectories of PS80 signals have the same general tendencies as for His signals, suggesting that they are both modulated by transient reactive radicals, which have paramagnetic properties.

**Figure 7:**
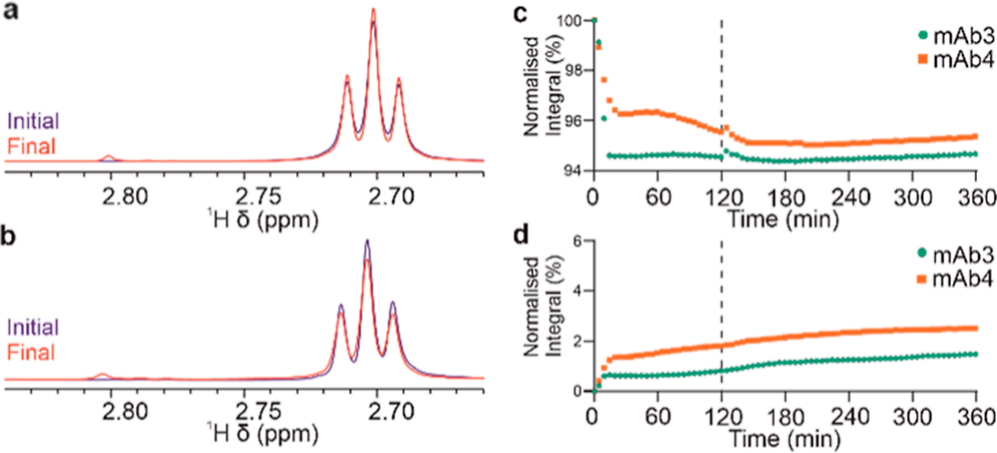
Degradation of Met excipient in mAb formulations. Appearance of a specific degradation signal in mAb3 (a) and mAb4 (b) with 20 mM Met. Evolution of Met Hγ signal (c, 2.70 ppm) and degradation signal (d, 2.80 ppm), with signal integrals normalized against the initial Met Hγ signal integral. Typical S/N for Met signals was >5000.

Finally, we looked at the evolution of Met signals for two formulations, where 20 mM Met was added as an excipient. In mAb3 and mAb4 samples with Met, a specific degradation signal appeared in both samples under UV illumination (Figure 7a,b), which was not present in samples without Met. Following UV illumination, the main signal from Met for both mAb3 and mAb4 formulations decreases considerably (Figure 7c) within the first 5–10 min of illumination, followed by a slower drop, while the signal from the degradation product rapidly increased (Figure 7d). As Met is usually added as a sacrificial antioxidant, it is likely that this initial degradation is caused by the reaction with the ROS formed from the oxygen initially dissolved in the formulation. The new signal likely arises from a specific Met oxidation product, the structure of which may be determined in the future by performing additional NMR experiments or by other orthogonal techniques.^42^

## Conclusions

Forced degradation studies of mAb formulations are useful at providing insights into the potential degradation time scales and mechanisms and inform where one must minimize exposure to the degradation stimulus during real-life scenarios. Reproducing forced degradation conditions may involve adding components that are not present normally, such as hydrogen peroxide, to speed up oxidation.^33,34^ Sometimes, the addition of probe molecules, such as TEMPOL, may be required for detecting radicals post-illumination.^4−6^ Adding non-native components to otherwise finely tuned formulation may, however, change its properties and, ideally, need to be avoided. Here, we performed forced UV degradation studies on mAb formulations without any such additives. Solutions were intrinsically photosensitized by a subtle balance of unknown factors, likely comprising mAbs themselves, the excipients such as His, PS80, and Met, and possible trace contaminants (metal cations, etc.). Use of in situ light-coupled NMR spectroscopy with real-time spectral reporting ensured that the effect of the appearance and disappearance of transient photoinduced reactive species could be monitored live, during and immediately after the illumination, and attributed exclusively to the effects of illumination. The characteristic lifetimes of these transient species and time scale of changes appear to be in the range of minutes to tens of minutes, meaning that ex situ analysis may miss them entirely. The paramagnetic nature of these transient radical species means that NMR signals from mAbs as well as excipient components become distorted, also hinting at the existence of complex interconverting chemical reaction pathways eventually consuming these transient species while also chemically modifying formulation components. Previously, we suggested that observing NMR signal intensities of mAb and excipients can reveal the instabilities in formulations;^37^ however, the presence of transient paramagnetic species induced by light significantly complicates such quantitative analysis due to additional signal broadening. Similarly, NMR experiments assessing the higher order structure of the end state of mAb after the illumination^4−6^ should wait for significant amount of time for the transient species to disappear to avoid paramagnetic distortions of the observable signals. It would be informative to explore directly the nature of these transient radicals using in situ light-coupled EPR spectroscopy, provided that suitable arrangements are made for strong UV illumination of the samples.

Here, we also present the results of conventional ex situ HP-SEC analysis of light-induced degradation of four mAbs in formulations with PS80 or Met as excipients, with the maximal UV dosage comparable between NMR and HP-SEC experiments (Figure S1 here and supporting Figure S6 in ref (38)). In line with our NMR observations, HP-SEC analysis revealed photodegradation of mAbs, with the rates and dominant pathways of degradation differing across formulations, suggesting that there is no clear and universal route to stabilization. Met is usually added as sacrificial antioxidant; here, in the presence of UV illumination, it generally reduced loss of monomer for mAb2, mAb3, and mAb4, according to ex situ HP-SEC analysis, predominantly via a decrease in the rate of aggregate formation. The presence of Met, however, caused maximal drop in observable NMR signal intensities for the same formulations, suggesting higher amounts of transient reactive radical species forming with Met itself noticeably degrading. As NMR and HP-SEC parameters reported on slightly different aspects of formulation degradation and were additionally affected differently by the presence of transient paramagnetic species, the profiles from ex situ and in situ experiments cannot be exactly matched but rather they need to be considered in combination, contributing to the same complex story from different viewpoints.

The complexity of the underlying light-driven transient processes revealed here by in situ analysis suggests that further research may be needed to characterize the elusive short-lived light-induced radicals. Importantly, NMR signal evolution continued for tens of minutes in the darkness following the initial illumination, indicating that once triggered, the chemical reactions leading to mAb degradation may persist for considerable amount of time. The analysis performed here was not looking at possible chemical modifications of mAbs following the illumination, due to a lack of spectral resolution of 1D spectra employed, but such chemical modifications cannot be excluded and would need to be monitored and explored in the future, for example, using NMR experiments for mAb fingerprinting such as the PROFILE or PROFOUND analysis.^40,41^

The design of the NMRtorch used here for the sample illumination with NMR detection means that different LEDs, or combinations, can be used,^38^ for example, to match ambient light in a specific environment or assess photodegradation at specific wavelengths.^8^ Light power is sufficient to achieve the dosage prescribed by ICH Q1B guidelines in less than 2 h, meaning that, in principle, NMRtorch can be adopted for routine photostability testing in situ. Any suitable existing or novel NMR experiments can then be used for the analysis of various aspects of photodegradation. As mAb photodegradation is light dosage dependent,^45^ i.e., a function of light intensity and illumination duration, accelerated photostability studies using intense light sources and shorter illumination durations may offer insights into photodegradation in a timely fashion. Specific photodegradation, such as Met and tryptophan oxidation,^33,34^ may also be detected in mAbs using multidimensional NMR spectroscopy.

## Supporting Information Available

Change of optical density of 2% quinine actinometrical control sample in the light chamber used for HPLC-SEC mAb UV stress studies; UV stress studies of mAb solutions analyzed by HP-SEC, percentage of HMW aggregates; UV stress studies of mAb solutions analyzed by HP-SEC, percentage of LMW fragments; typical behavior of His H^δ^ signal during and after UV illumination in the mAb1 formulation in His buffer; T_1_ relaxation of His side chain protons before and after UV-A illumination; T_2_ relaxation of His side chain protons before and after UV-A illumination; and translational diffusion coefficients measured for His H_δ_ and H_ε_ protons before and after UV-A illumination (PDF)
