# Supplementary material for: Assessing Photostability of mAb Formulations In Situ Using Light-Coupled NMR Spectroscopy
Source: Anal Chem. 2024 Jun 7;96(24):9935–43. doi: 10.1021/acs.analchem.4c01164 (PMC11190875; doi:10.1021/acs.analchem.4c01164)
Supplement: Supplementary file 1 — ac4c01164_si_001.pdf [file ac4c01164_si_001.pdf]

Supporting Information

Assessing photo-stability of mAb formulations *in situ* using light-coupled NMR spectroscopy

Jack E. Bramham,[1] Yujing Wang,[2] Stephanie A. Moore,[2] and Alexander P. Golovanov[1*]

[1] Department of Chemistry, School of Natural Sciences, Faculty of Science and Engineering, The University of Manchester, Manchester M1 7DN, U.K.

[2] Dosage Form Design & Development, BioPharmaceutical Development, R&D, AstraZeneca, Cambridge CB21 6GH, U.K.

* Correspondence to: a.golovanov@manchester.ac.uk

TABLE OF CONTENTS

Figure S1: Change of optical density of 2% quinine actinometrical control sample at 400 nm following UV-A exposure in the light chamber used for HPLC-SEC mAb UV stress studies.

Figure S2: UV stress studies of mAb solutions analysed by HP-SEC.

Figure S3: UV stress studies of mAb solutions analysed by HP-SEC.

Figure S4: An example of typical behaviour of histidine H^{δ} signal during and after UV illumination, in the mAb1 formulation in His buffer.

Figure S5: T_1 relaxation of Histidine sidechain protons before and after UV-A illumination.

Figure S6: T_2 relaxation of Histidine sidechain protons before and after UV-A illumination.

Figure S7: Translational diffusion coefficients (D_L) measured for His buffer sidechain Hδ and Hε protons before and after UV-A illumination.

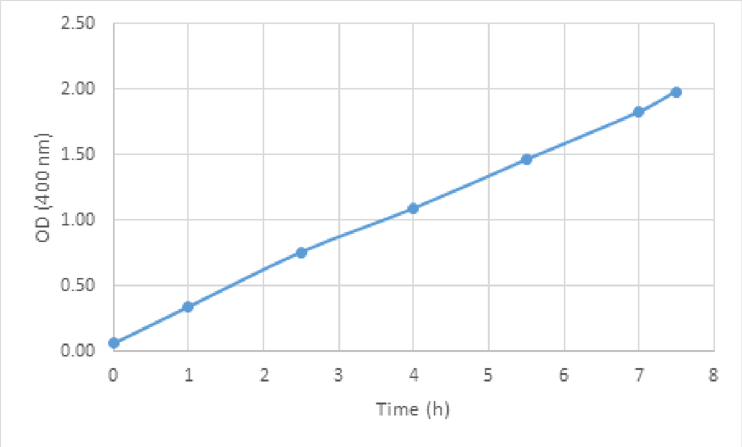

Figure S1. Change of optical density of 2% quinine actinometrical control sample at 400 nm following UV-A exposure in the light chamber used for HP-SEC mAb UV stress studies. The illumination dosage prescribed by the International Council for Harmonization of Technical Requirements for Pharmaceuticals for Human Use (ICH) Q1B (https://database.ich.org/sites/default/files/Q1B%20Guideline.pdf) for photostability testing requires the chemical actinometer sample (quinine) to change its optical absorbance at 400 nm (OD_{400}) by more than 0.5. Here the illumination dosage at 7.5 hours, leading to OD_{400} of around 2, corresponds to approximately 2 h dosage in the NMRtorch setup, also leading to OD_{400} of around 2, see Supplementary Figure 6 in (Bramham and Golovanov, *Commun Chem* **5**, 90 (2022). https://doi.org/10.1038/s42004-022-00704-5). The overall UV dosage used here in HP-SEC and NMRtorch experiments therefore match, both meeting ICH Q1B requirements.

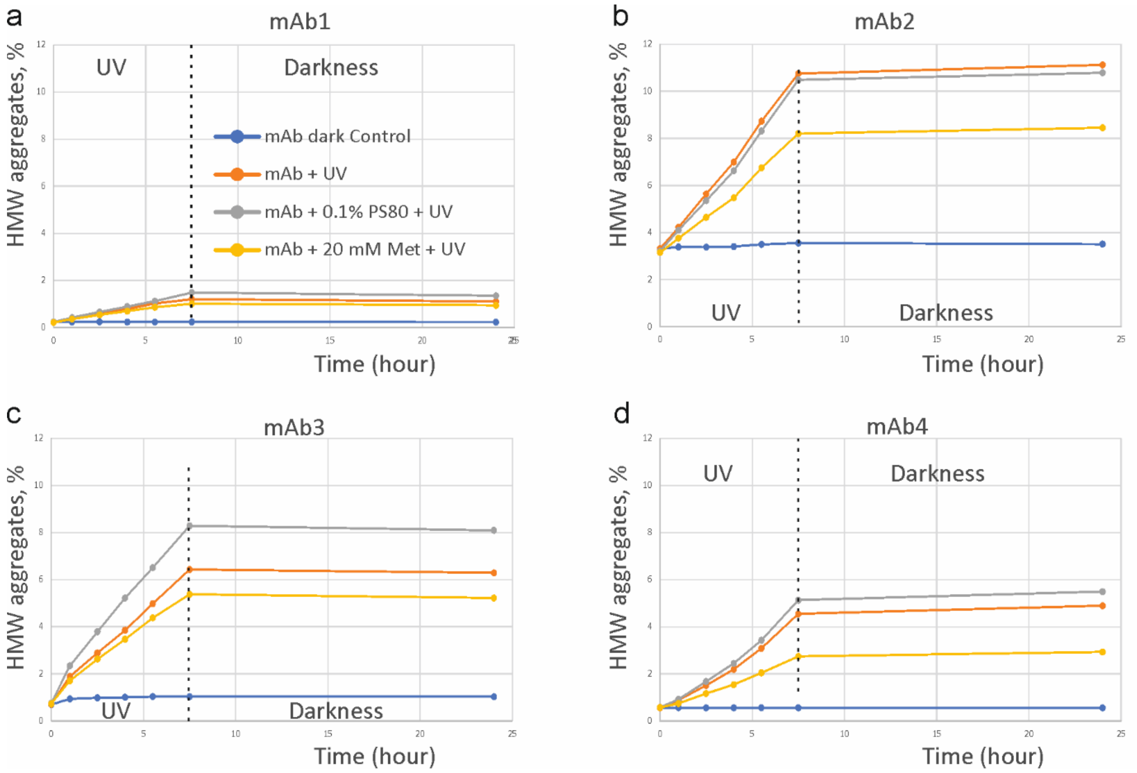

Figure S2. UV stress studies of mAb solutions analysed by HP-SEC. Percentage of higher molecular weight (HMW) soluble aggregates is shown for mAb1 (a), mAb2 (b), mAb3 (c) and mAb4 (d). Dark Control samples were kept in UV chamber but wrapped in aluminium foil to prevent UV exposure. All solutions contained 20 mM His buffer pH 5.5, without or with excipients added as shown. After 7.5 hours UV light was switched off, with another aliquot taken for analysis at 24 hours to explore further degradation in the darkness. Changes of <0.2% are not considered significant due to inherent limitations of the HP-SEC analysis.

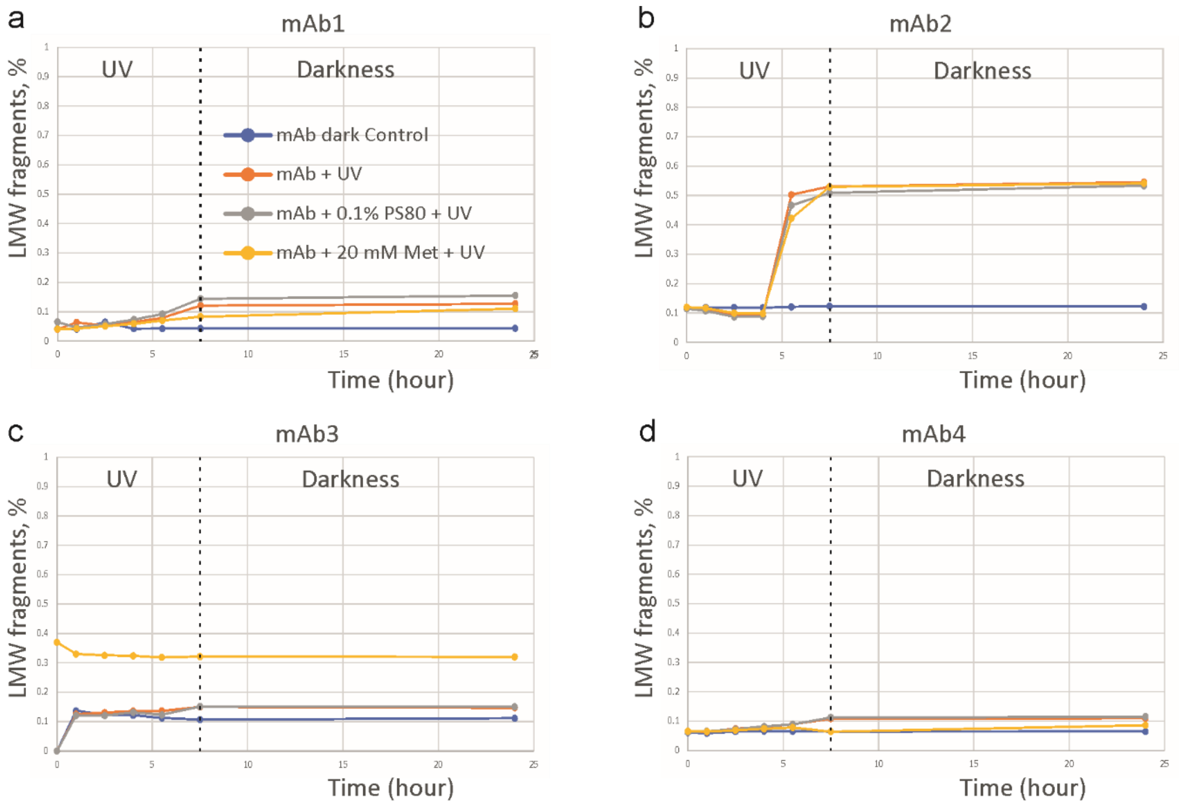

Figure S3. UV stress studies of mAb solutions analysed by HP-SEC. Percentage of lower molecular weight (LMW) fragments is shown for mAb1 (**a**), mAb2 (**b**), mAb3 (**c**) and mAb4 (**d**). Dark Control samples were kept in UV chamber but wrapped in aluminium foil to prevent UV exposure. All solutions contained 20 mM His buffer pH 5.5, without or with excipients added as shown. After 7.5 hours UV light was switched off, with another aliquot taken for analysis at 24 hours to explore further degradation in the darkness. Changes of <0.2% are not considered significant due to inherent limitations of the HP-SEC analysis.

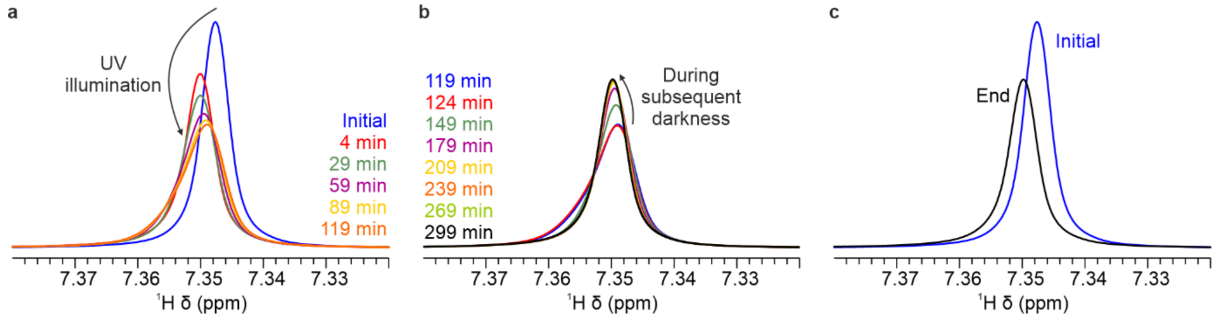

Figure S4. An example of typical behaviour of histidine H^{δ} signal during and after UV illumination, in the mAb1 formulation in His buffer. NMR spectra during UV illumination (**a**), during darkness following illumination (**b**), and comparing the initial and end points (after illumination and dark waiting period) (**c**). Similar behaviour observed for all mAbs and formulations. Curved arrows depict the movement trajectory of signal maxima.

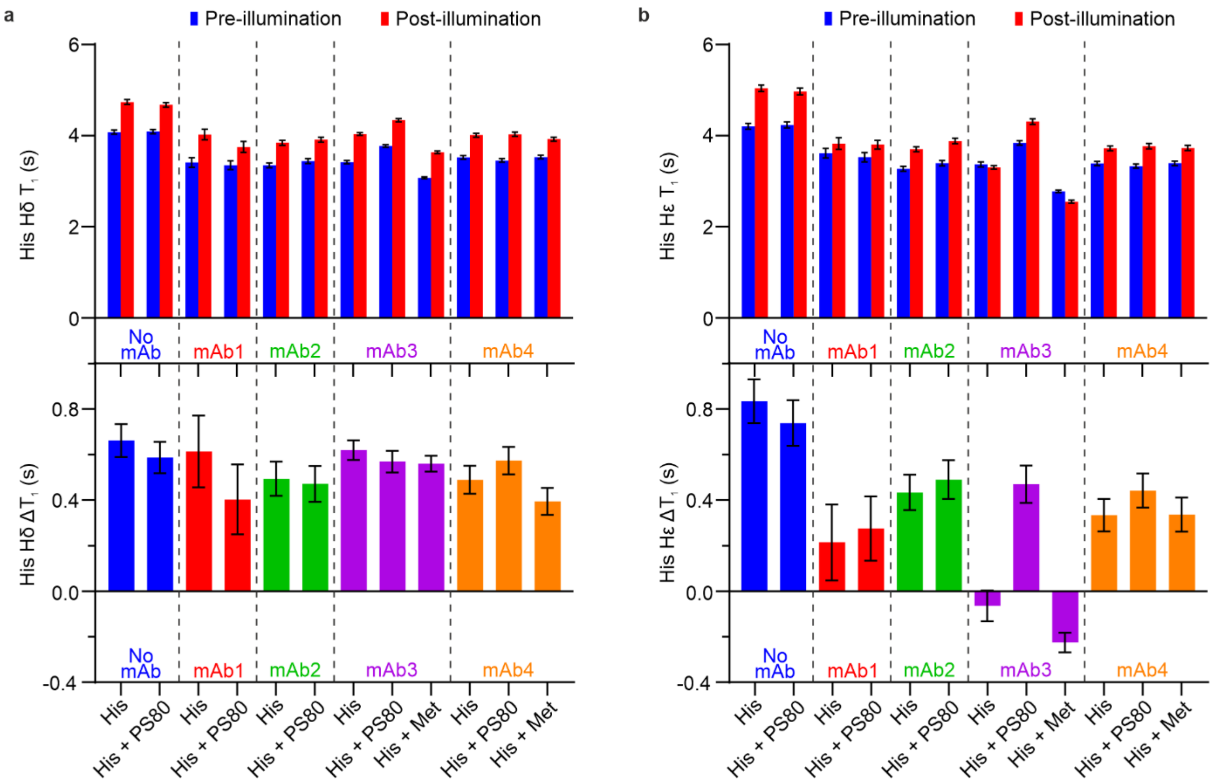

Figure S5. T_1 relaxation of Histidine sidechain protons before and after UV-A illumination followed by the relaxation in the dark. Apparent (T_1) and changes (ΔT_1) relaxation times for Hδ (**a**) and Hε (**b**). Error bars from 95% confidence intervals from relaxation time fitting. Here, T_1 is systematically longer after the illumination, consistent with oxygen initially present in the sample been consumed. The only marginally significant exception is decreased relaxation time T_1 of Hε of His for mAb3 formulation in the presence of Met.

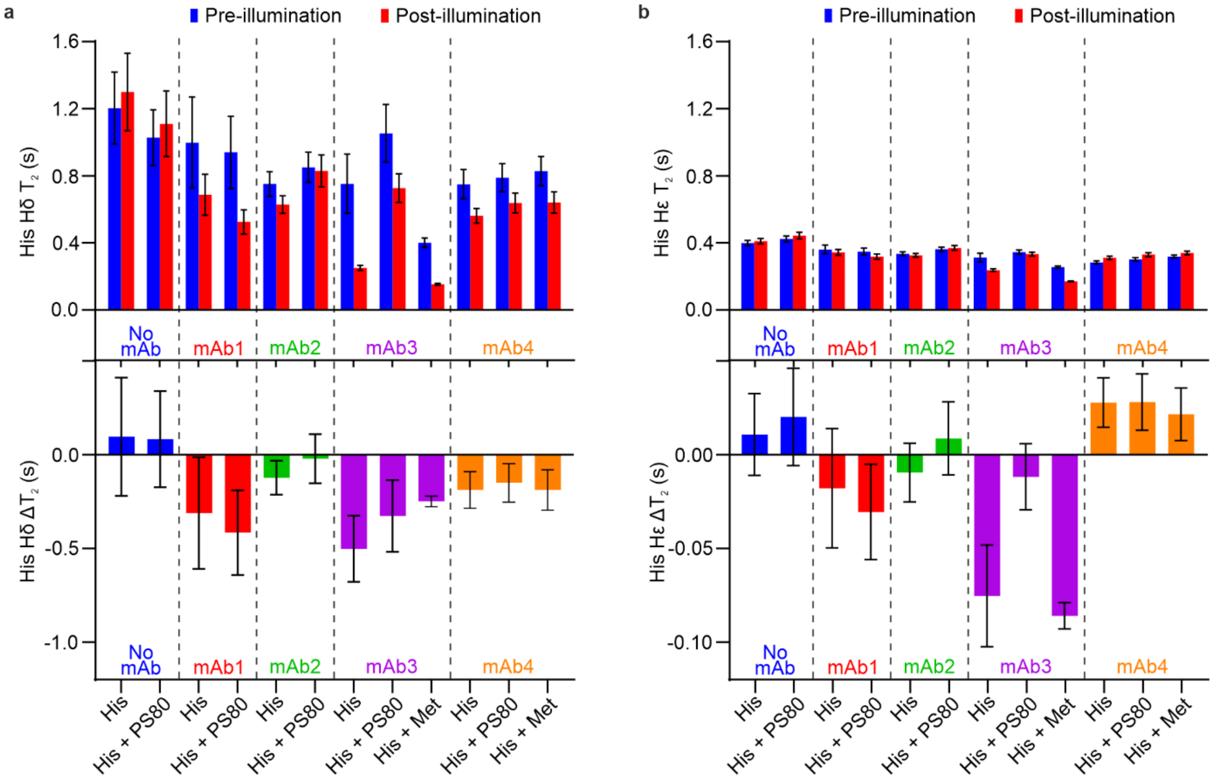

Figure S6. T_2 relaxation of Histidine sidechain protons before and after UV-A illumination followed by the relaxation in the dark. Apparent (T_2) and changes (ΔT_2) relaxation times for Hδ (**a**) and Hε (**b**). Error bars from 95% confidence intervals from relaxation time fitting. Here, T_2 of His signals after the illumination mostly showed a borderline tendency to decrease (ie, signal broaden) for the majority of mAb formulations, which may be caused by increased aggregation and self-association of mAbs.

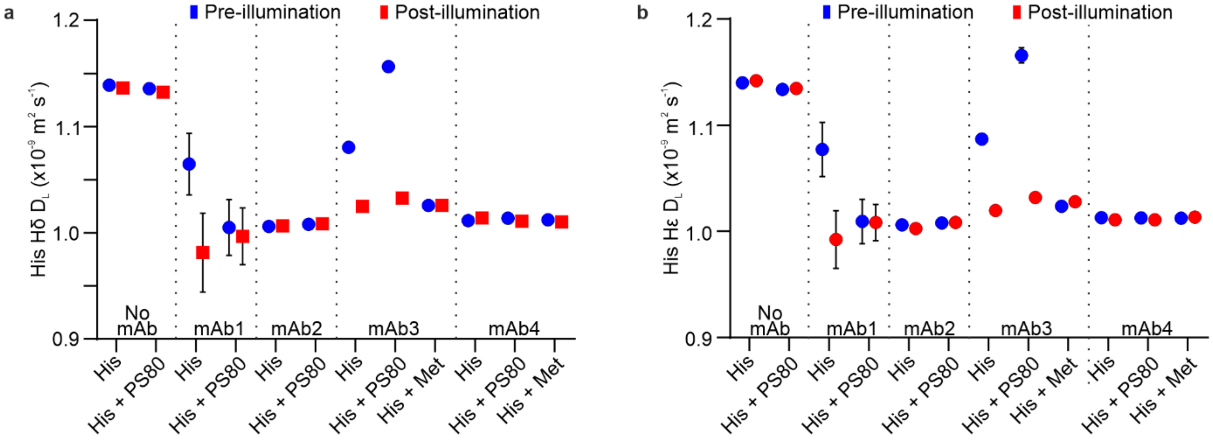

Figure S7. Translational diffusion coefficients (D_L) measured for His buffer sidechain Hδ and Hε protons before and after UV-A illumination followed by the relaxation in the dark. Apparent diffusion coefficients for Hδ (**a**) and Hε (**b**). Error bars from 95% confidence intervals from diffusion coefficient fitting. For the majority of mAb solutions the changes in D_L were not significant, except for bispecific mAb3 formulations with His or His+PS80, where D_L was decreased significantly post illumination, suggesting a light-induced increase in formulation viscosity.
